# A Feedback Loop Involving MicroRNA-150 and MYB Regulates VEGF Expression in Brain Microvascular Endothelial Cells After Oxygen Glucose Deprivation

**DOI:** 10.3389/fphys.2021.619904

**Published:** 2021-03-17

**Authors:** Song Zhang, Anqi Chen, Xiaolu Chen

**Affiliations:** ^1^Department of Dermatology, Union Hospital, Tongji Medical College, Huazhong University of Science and Technology, Wuhan, China; ^2^Department of Neurology, Union Hospital, Tongji Medical College, Huazhong University of Science and Technology, Wuhan, China

**Keywords:** miRNA-150, VEGF – vascular endothelial growth factor, OGD – oxygen/glucose-deprivation, MYB, angiogeneisis

## Abstract

Vascular endothelial growth factor (VEGF) plays a pivotal role in regulating cerebral angiogenesis after stroke. Meanwhile, excessive VEGF expression induces increased microvascular permeability in brain, probably leading to neurological deterioration. Therefore, the appropriate level of VEGF expression is significant to the recovery of brain exposed to stroke. In this work, we demonstrate that microRNA-150 (miR-150) and its predicted target MYB form a negative feedback loop to control the level of post-stroke VEGF expression. Repression of MYB leads to decreased expression of miR-150 in brain microvascular endothelial cells (BMVECs) exposed to oxygen glucose deprivation (OGD), thus miR-150 was predicted to be down-regulated by MYB. Moreover, MYB was confirmed to be a direct target of miR-150 by using dual luciferase reporter assay. In our previous work, we have validated VEGF as another direct target of miR-150. Therefore, MYB participates in regulation of VEGF via miR-150 under OGD, forming a feedback loop with miR-150. We also find that high levels of miR-150 inhibitors combined with MYB silence contribute to further enhancement of VEGF expression in BMVECs in response to OGD. These observations suggest that the feedback loop comprised of miR-150 and MYB, which is a pivotal endogenous epigenetic regulation to control the expression levels of VEGF in BMVECs subjected to OGD.

## Introduction

Fostering compensatory angiogenesis contributes to the restoration of blood supply and provides one of the most promising therapeutic strategies for cerebral ischemia (Hatakeyama et al., 2020). Among numerous endogenous angiogenetic regulators, Vascular endothelial growth factor (VEGF) plays a leading role in governing cerebral angiogenesis (Giacca and Zacchigna, 2012). However, excessive VEGF expression and oxygen glucose deprivation (OGD) induce increased microvascular permeability which could further damage the blood brain barrier (BBB) (Kaur and Ling, 2008; Kunze and Marti, 2019), consequently resulting in expanded brain edema after stroke and neurological deterioration (Geiseler and Morland, 2018). Therefore, right level of VEGF is critical for functional recovery following cerebral ischemia and hence, it is significant to explore novel mechanisms that can modulate VEGF.

MicroRNAs (miRNAs) are a family of non-coding, endogenous small RNAs (19–23 nucleotides) that control gene expression at the post-transcriptional level. Multiple biological processes including cell proliferation, differentiation, tumor progression and also angiogenesis could be controlled by miRNAs (Yin et al., 2015; Tiwari et al., 2018). Among these miRNAs, miR-150 was found to be down-regulated in the blood and brain of rats subjected to Middle Cerebral Artery Occlusion (Choi et al., 2016). Our previous studies have suggested that miR-150 is expressed in brain microvascular endothelial cells (BMVECs) of rats and that miR-150 could indeed suppress VEGF expression directly by binding to 3′UTR of VEGF mRNA (He et al., 2016). Meanwhile, inhibition of miR-150 promoted cerebral angiogenesis and functional rehabilitation after stroke in rat (Fang et al., 2016; He et al., 2016). The transcription factor MYB, a proto-oncogene, which is highly expressed in hematopoietic progenitors as well as leukemia and certain solid tumors, can also be capable of regulating cell proliferation, differentiation and angiogenesis (Liu et al., 2018; Yang et al., 2018; Deng et al., 2020). In this study, we observed impaired tube formation of BMVECs following inhibition of MYB under OGD. Interestingly, miR-150 expression decreased and corresponding VEGF expression was augmented in this circumstance. Not surprisingly, functional deficiency of MYB was associated with BMVECs tube forming capacity and modulation of VEGF expression. Thus, this finding highlighted a novel strategy involving MYB for controlling the expression level of VEGF through regulation of miR-150. As a typical target of miR-150, MYB is also negatively controlled by miR-150, therefore, maintaining a balanced state with miR-150 for VEGF regulation.

In light of the above findings, we hypothesized theoretically that repression of MYB was able to further increase VEGF expression in the presence of higher levels of miR-150 inhibitors under OGD and may subsequently induce significant angiogenesis as well as increased vascular permeability which can be disastrous. In this study, we provide evidence to validate that miR-150 directly represses expression of MYB in BMVECs, and MYB in turn promotes miR-150. Furthermore, enforced inhibition of miR-150 alone using miR-150 inhibitors failed to further enhance VEGF expression in BMVECs in response to OGD but when MYB was also inhibited using MYB siRNA, VEGF expression was further enhanced. Hence, our study identifies a negative feedback loop involving miRNA-150 and MYB that maintains the homeostatic expression of VEGF in BMVECs under OGD.

## Materials and Methods

### Experimental Animals

We purchased Sprague-Dawley rats from Beijing HFK Bioscience Co. Ltd. (Beijing, China). Rats were bred in the animal facility under specific pathogen-free (SPF) conditions and they were age- and weight-matched when used in experiments. Animal experiments was performed in the USUHS laboratory animal facility. The protocol used in these experiments had been approved by the USUHS Institutional Animal Care and Use Committee.

### Culturing of BMVECs and 293T Cells

BMVECs were isolated from Sprague-Dawley rats (*n* = 5, 3–5 weeks of age) according to protocols in our previous studies (He et al., 2013). Briefly, rats were swabbed and sacrificed, and their brains were collected. Then, white matter, brain stem, surface vessels, and leptomeninges were carefully removed. The isolated cerebral cortices were put into ice-cold phosphate-buffered saline (PBS), minced into small pieces, and homogenized. The homogenates were centrifuged at 500 × *g* for 5 min at 4°C. The pellet was re-suspended in 20% bovine serum albumin (BSA) and centrifuged at 1,000 × *g* for 20 min at 4°C. The micro-vessels in the lower layer were transferred to a new tube. After washing with PBS one time, the micro-vessel pellets were digested by using 0.1% collagenase II/dispase and 1,000 U/ml DNase I at 37°C for 1 h. After centrifugation at 500 × g for 5 min (4°C), the micro-vessel pellets were re-suspended in 10 ml of M131 medium (Invitrogen, United States) supplemented with microvascular growth supplement (Invitrogen, United States), 100 U/ml penicillin and streptomycin. The cell suspension was seeded into a 75-cm^2^ flask, incubated at 37°C in humidified 5% CO_2_/95% air, and three to six passages of BMECs were used. Also, Human embryonic kidney 293T (HEK293T) cells were grown in Dulbecco’s Modified Eagles Medium (DMEM) with 10% fetal bovine serum (FBS).

### Oxygen Glucose Deprivation and Reoxygenation

In accordance with a previous report with some modifications, cells were exposed to OGD for 2, 4, 6 h and then resumed with the oxygen and glucose supply for 24 h. In brief, the culture medium was replaced with a hypoxic medium previously saturated for 20 min with 95% N_2_ and 5% CO_2_, which contains 116 mM NaCl, 5.4 mM KCl, 0.8 mM MgSO_4_, 26.2 mM NaHCO_3_, 1 mM NaH_2_PO_4_, 1.8 mM CaCl_2_, and 0.01 mM glycine. Hypoxic conditions were maintained by using a hypoxia chamber (temperature 37°C, atmosphere 95% N_2_ and 5% CO_2_). Re-oxygenation was achieved by returning to normoxic conditions (37°C in a humidified 5% CO_2_ atmosphere).

### Observation of Tube Formation

Basement membrane matrix (Matrigel, BD Biosciences) was thawed at 4°C, and 300 μl was used to coat each well of a 24-well plate. The plate was incubated for 30 min at 37°C to polymerize the Matrigel. BMVECs (5 × 10^4^ cells/100 μl) were suspended in M131 containing MGS, plated into a Matrigel-coated well, and incubated for 24 h at 37°C. Tube length was quantitatively determined at ×40 magnifications from pictures captured. Each sample was examined in three randomly selected fields, and the examination was repeated three times.

### Oligonucleotides and Transfection

miR-150 mimics, inhibitors, and the corresponding negative control molecules were purchased from RIBOBIO. A mixture of Lipofectamine2000 transfection agent (RIBOBIO, China) in Opti-MEM medium and miR-150 inhibitors, mimics or the corresponding negative control molecules in Opti-MEM medium was prepared for 30 min at 37°C according to the manufacturer’s instructions. MiR-150 inhibitors were transfected with this solution in BMVECs or 293T at a final concentration of 50, 100, 200, 300, or 500 nM while mimics and negative control molecules in BMVECs reached a common concentration. 24 h after transfection, cells were used for further processing.

### siRNA Transfection

Lentivirus was commercially obtained and titration was performed according to the directions of the manufacturer (Genechem, China). siRNA-mediated knockdown of MYB (Gene ID: 498982) in BMVECs was achieved by transfection of lentivirus using a siRNA oligonucleotide kit according to the kit instruction. A commercially procured non-targeting siRNA from the same corporation was used as a negative control. The incorporation of siRNAs into BMVECs was detected by fluorescence microscopy. Western blot assay was used to identify the expression of MYB in siRNA-transfected BMVECs by employing the rabbit anti-MYB antibody (Abcam, 1:200) while quantitative real-time reverse transcriptase-polymerase chain reaction (qRT-PCR) was performed to test the levels of mRNA.

### RNA Isolation and qRT-PCR

Total RNA was isolated from the harvested cells by Trizol Reagent (Invitrogen, United States) according to the manufacturer’s instructions. cDNA was synthesized via the PrimeScript^®^ RT Master Mix Perfect Real Time Kit (TAKARA BIO INC., Japan) and amplified with a SYBR^®^ Premix Ex Taq^TM^ Kit (TAKARA BIO INC., Japan). Primer sequences are as follows: forward 5′-TCTCCCAACCCTTGTACCAGTG-3′ for miR-150 and 5′-CTCGCTTCGGCA GCACA-3′ for U6; forward 5′- GCGGGCTGCTGCAATG-3′ and reverse 5′-TGCAACGCGAGTCTGTGTTT-3′ for VEGF; forward 5′-TGGAATCCTGTGGCATCCATGAAAC-3′ and reverse 5′-TAAAACGCAG CTCAGTAACAGTCCG-3′ for β-actin. The comparative threshold cycle (CT) method was employed to calculate the target genes relative to the endogenous control. For the miRNAs, the results were normalized with the endogenous U6 control while for the mRNAs, β-actin was used as the endogenous control. All reactions were performed in triplication.

### Western Blot Analysis

Cells were harvested and lysed in lysis buffer (50 mM Tris–HCl, 150 mM NaCl, 1% Non-idet P-40, 0.5% sodium deoxycholate, 0.1% SDS, and 1 mM phenylmethylsulfonyl fluoride). A Bio-Rad protein assay kit was used to determine protein concentrations, with bovine serum albumin acting as a reference. Samples containing 30 μg of protein were put on 10% SDS-polyacrylamide gels and electrophoretically transferred to nitrocellulose membranes. Membranes were incubated with specific primary antibodies including rabbit anti-VEGF antibody (Abcam, 1:1,000), rabbit anti-MYB antibody (Abcam, 1:400) and rabbit anti-β-actin antibody (Abcam, 1:2,000), then membrans were incubated with horseradish peroxidase-conjugated secondary antibody (Sigma, 1:10,000). Proteins were evaluated using a SuperSignal West Pico chemiluminescence kit (Thermo Scientific, United States).

### ELISA

100 μl of medium was collected from different groups of BMVECs, and the protein expression levels of VEGF were detected by employing a rat-specific VEGF ELISA kit (USCNK Life Science Inc., China) according to the manufacturer’s instructions.

### Dual-Luciferase Reporter Assay

Dual-luciferase reporter assay was employed to determine whether miR-150 directly targeted MYB. Region that contained two predicted binding sites of miR-150 was cloned into the pmiR-RB-REPORT TM luciferase reporter plasmid (GENECHEM, China) to construct the MYB WT reporter plasmid. pmiR-MYB-vector (containing none of MYB 3′-UTR binding sites), pmiR-MYB-mut1 (containing one of mutant MYB 3′-UTR binding sites), pmiR-MYB-mut2 (containing another mutant MYB 3′-UTR) and pmiR-MYB-mut1mut2 (containing both mutant MYB 3′-UTR binding sites) were also constructed. BMVECs were plated into 24-well plates and co-transfected with 500 ng of different pmiR-MYB reporter plasmids and 100 nM miR-150 mimic or mimic negative control with lipofectamine2000. Cells were harvested after 48-h transfection and luciferase activities were detected by employing a dual-luciferase reporter assay system (Promega, China) according to manufacturer instructions.

### Statistics

Statistical Package for the Social Sciences (SPSS 13.0, United States) software was used to analyze the data and the two-tailed Student’s *t*-test was performed for significance assessment. *P*-values of <0.05 were considered statistically significant.

## Results

### MYB Is Up-Regulated in BMVECs and Modulates BMVECs Tube Formation After OGD

**FIGURE 1:**
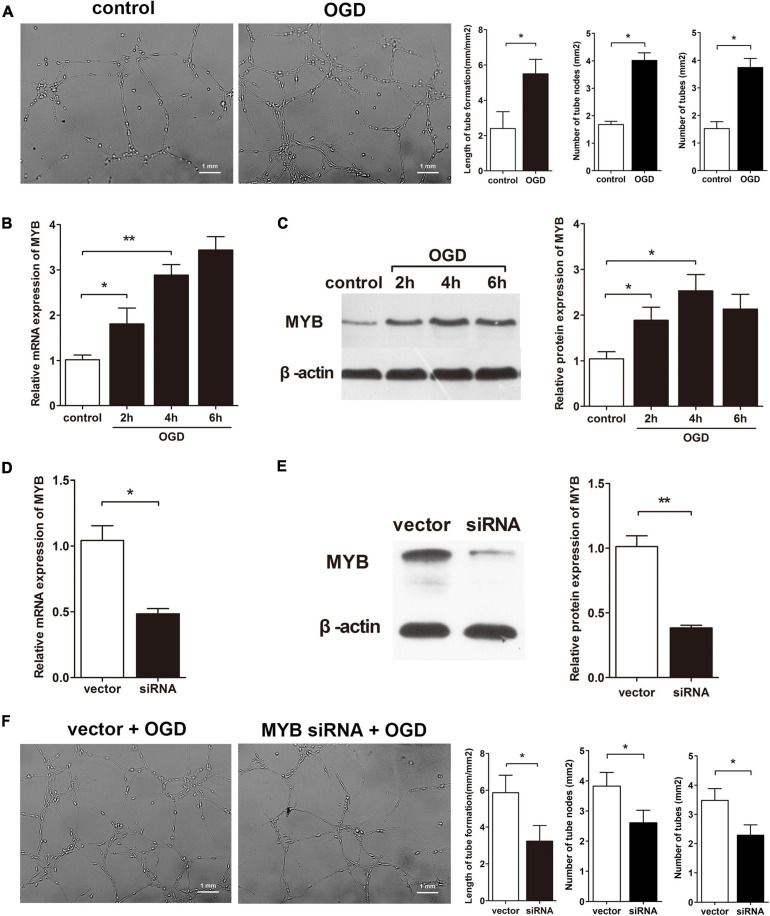
MYB is up-regulated in BMVECs and modulates BMVECs tube formation after OGD. **(A)** BMVECs were plated into martrigel for 6 h under OGD, and then cultured for 24 h under normal condition. Then capillary-like tubes were microscopically observed, pictures displaying randomly selected fields of vision were captured and quantitatively measured by employing Image J software package. Bar = 1 mm. BMVECs were exposed to OGD for different time periods (2, 4, or 6 h), expression of MYB was detected by **(B)** qRT-PCR and **(C)** western blot analysis. **(D)** qRT-PCR and **(E)** western-blotting analysis of MYB expression at mRNA and protein levels in BMVECs transfected with lentivirus carrying MYB siRNA or siRNA control (vector). **(F)** BMVECs were transfected with MYB siRNA or vector for 4 days and then cells were transplanted into matrigel under OGD for 6 h before the next 24 h culture. Length and number of tubes were calculated as previously described. Bar = 1 mm. Data are presented as mean ± SD, *n* = 5 of three independent experiments. *p < 0.05; **p < 0.01 versus the control group.

Evidences have revealed MYB as a transcription factor involved in endothelial cells proliferation and migration which are key processes involved in angiogenesis (Li et al., 2013); this phenomenon can also be observed in BMVECs during OGD as our group has identified (He et al., 2013). Since the ability of BMVECs to form capillary-like structures also plays a pivotal role in cerebral angiogenesis after OGD, tube formation by BMVECs can be studied. We manipulated BMVECs under OGD or normoxia (control), identifying amplified potency of tube formation by BMVECs under OGD compared to control (Figure 1A). To assess the change in MYB, BMVECs were exposed to OGD for different time periods (2, 4, or 6 h), then MYB in BMVECs was detected by qRT-PCR and western blotting. As expected, mRNA and protein levels of MYB were markedly up-regulated after OGD (Figures 1B,C) along with the enhancement of VEGF expression which was observed in our previous work. In the interest of contribution of MYB on BMVECs related angiogenesis, we transfected BMVECs with lentivirus carrying siRNA against MYB (MYB siRNA) or siRNA control (vector), then qRT-PCR and western-blotting were performed to determine the effect of lentivirus. MYB expression in BMVECs transfected with siRNA significantly decreased at both mRNA and protein level (Figures 1D,E). BMVECs transfected with siRNA or vector under OGD were then plated on matrigel and cultured for 24 h, the length of capillary-like structures was calculated subsequently. Decreased length and number of capillary-like structures could be observed in groups of BMVECs transfected with MYB siRNA in comparison with vector groups (Figure 1F), suggesting a promoting effect of MYB on BMVECs involved angiogenesis.

### Repression of MYB Up-Regulates VEGF Expression but Down-Regulates miR-150 in BMVECs Subjected to OGD

**FIGURE 2:**
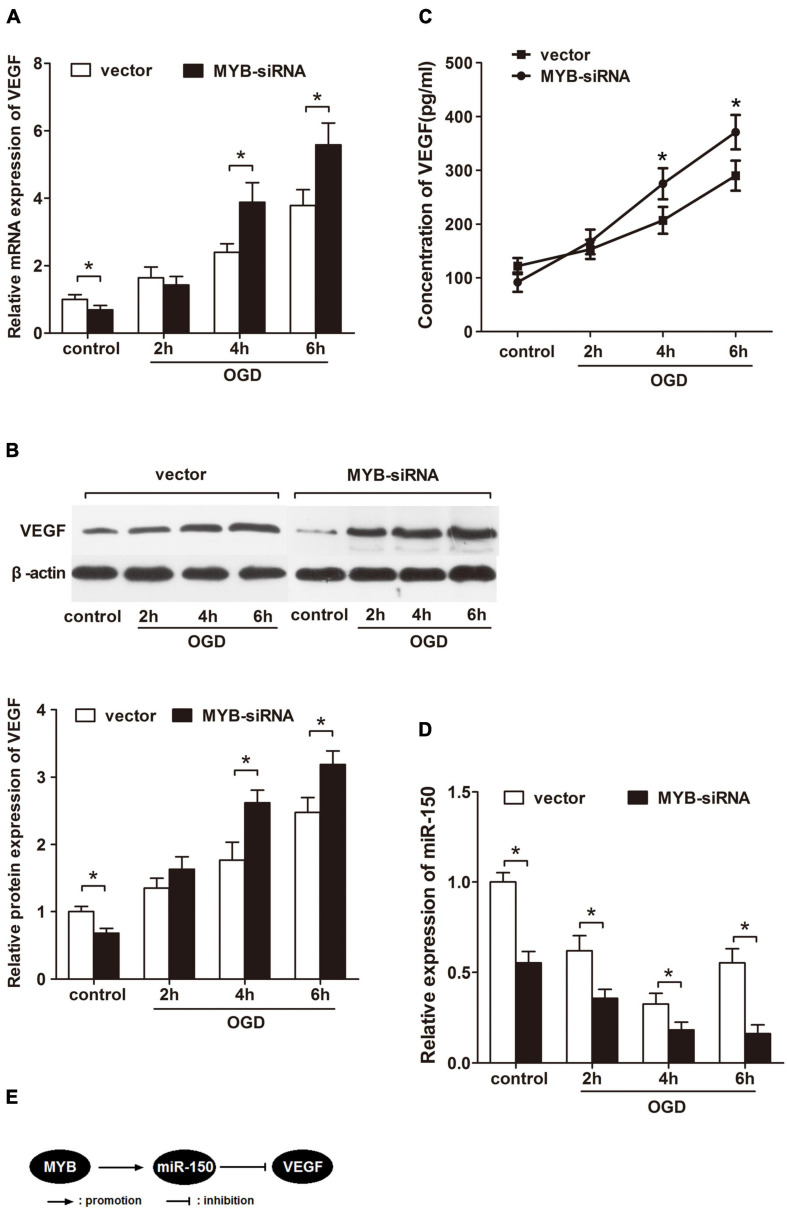
Repression of MYB up-regulates VEGF expression but down-regulates miR-150 in BMVECs subjected to OGD. BMVECs were transfected with MYB-siRNA or vector before subjecting to OGD for 2, 4, and 6 h, then re-perfused for 24 h. **(A)** qRT-PCR was performed to determine the expression of VEGF mRNA and **(B)** western blot analysis of protein levels in treated BMVECs was also carried out. The ratio of VEGF/β-actin in vector transfected group under normoxia was set to 1.0, and then values of other groups were calculated accordingly. **(C)** Concentration of VEGF in the medium was detected by ELISA. **(D)** Expression of miR-150 in the similarly treated BMVECs were detected by employing qRT-PCR. **(E)** A schematic diagram showing regulation of VEGF in BMVECs by MYB via miR-150 under OGD. Data are presented as mean ± SD, *n* = 5 of three independent experiments. *p < 0.05 versus the control group.

Since VEGF possesses a paramount role in cerebral angiogenesis after ischemia, whether the change in MYB expression has an influence on VEGF needs to be further explored. With this aim, MYB siRNA was transfected into BMVECs before exposing them to OGD for different time periods (2, 4, and 6 h). We next employed qRT-PCR to measure mRNA expression of VEGF in BMVECs under OGD. Intriguingly, knockdown of MYB induced more VEGF mRNA after OGD compared to vector group (Figure 2A). Consistent with the elevation of VEGF mRNA expression, VEGF protein level was also enhanced as measured by western-blotting (Figure 2B) and ELISA (Figure 2C) following inhibition of MYB under OGD. Our previous study has demonstrated VEGF as a direct target of miR-150, therefore, we harbored the idea that enhancement of VEGF expression was probably due to the enforced suppression of miR-150. Results from qRT-PCR revealed a continuous reduction in miR-150 expression in BMVECs until 6 h at which it decreased to the lowest point following MYB siRNA transfection whereas miR-150 level rebounded after introduction of vector at the same time point (Figure 2D), confirming the regulatory role of MYB on the expression of miR-150. Taken together, these data confirmed the regulatory role of MYB on VEGF levels through modulating miR-150 in BMVECs subjected to OGD (Figure 2E).

### MYB Is a Direct Target of miR-150 in BMVECs

**FIGURE 3:**
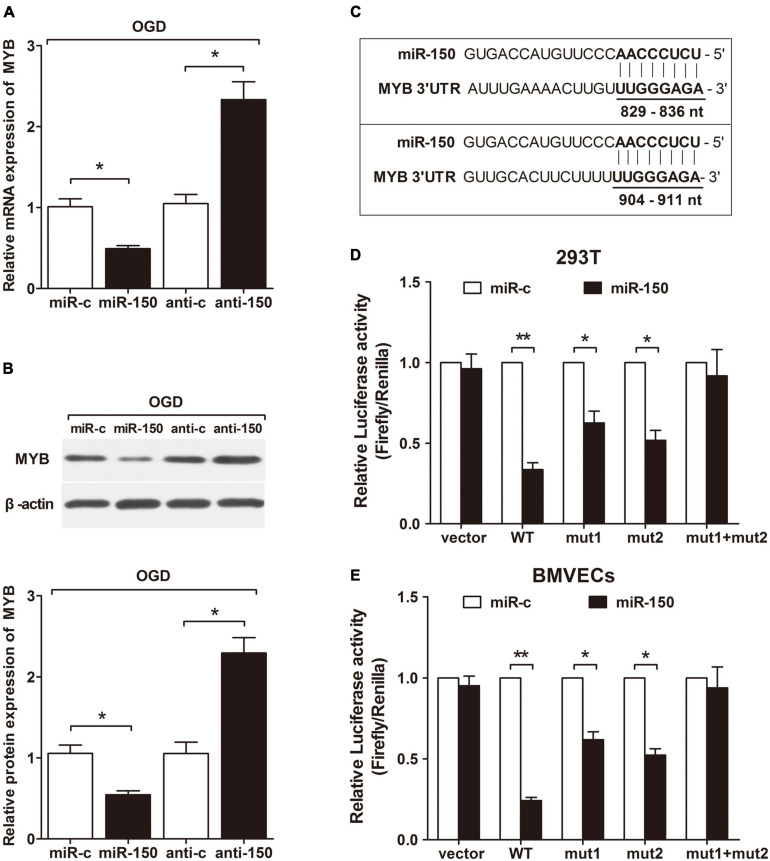
MYB is a direct target of miR-150 in BMVECs. BMVECs were transfected with miR-150 mimics (miR-150), miR-150 mimics control (miR-c), miR-150 inhibitor (anti-150) or miR-150 inhibitor control (anti-c) and then manipulated under OGD for 6 h. **(A)** qRT-PCR and **(B)** western blotting were performed to determine the mRNA and protein expressions. **(C)** Prediction of two binding sites (position 829–836 nt and 904–911 nt of MYB 3′UTR) of miR-150 in the 3′UTR of MYB mRNA. Region that contained these two binding sites was cloned into a luciferase reporter to construct the MYB WT reporter plasmid. **(D,E)**. Reporters containing mutations of either or both predicted binding sites of miR-150 were also constructed as MYB mut1, mut2, and mut1mut2. Vector indicated the reporter containing neither of the target sites. Cells were co-transfected with each constructed luciferase reporter and miR-150 mimics or mimics’ control. The relative luciferase activity was detected in BMVECs and 293T cells. Values were calculated by Firefly luciferase activity/renilla luciferase activity. Data are presented as mean ± SD, *n* = 5 of three independent experiments. *p < 0.05; **p < 0.01 versus the control group.

To explore the effect of miR-150 on endogenous MYB expression in BMVECs subjected to OGD, we employed qRT-PCR and western-blotting and observed that miR-150 mimic transfected BMVECs displayed a decreased MYB expression at both mRNA (Figure 3A) and protein level (Figure 3B) while an increased expression was seen in the presence of anti-miR-150. We searched for potential targets of miR-150 by using four target prediction software programs (TargetScan, miRanda, miRDB, and RNAhybrid). All these programs predicted MYB mRNA as the potential target of miR-150 and there were two classic miR-150 target seed sequences located in MYB 3′UTR (Figure 3C). To verify this prediction, we cloned complete 3′UTR and mutations of two different predicted binding sites on 3′UTR into luciferase reporter construct. Then miR-150 mimic and different reporters were co-transfected into 293T cells and BMVECs. Reporter assays revealed that miR-150 repressed luciferase activity driven by complete 3′UTR as well as either of both mutated 3′UTR, only both mutations of predicted miR-150 binding sites together abrogated miR-150 induced inhibition of luciferase expression (Figures 3D,E). These results suggest that miR-150 can strongly inhibit MYB expression through either of the two predicted target seed sequences in the 3′UTR. Taken together, these data validate that MYB is a direct target of miR-150, therefore suggesting that MYB and miR-150 form a negative regulatory loop in BMVECs exposed to OGD.

### Repression of MYB Is Required for miR-150 Inhibitor Mediated Further Enhancement of VEGF in BMVECs During OGD

**FIGURE 4:**
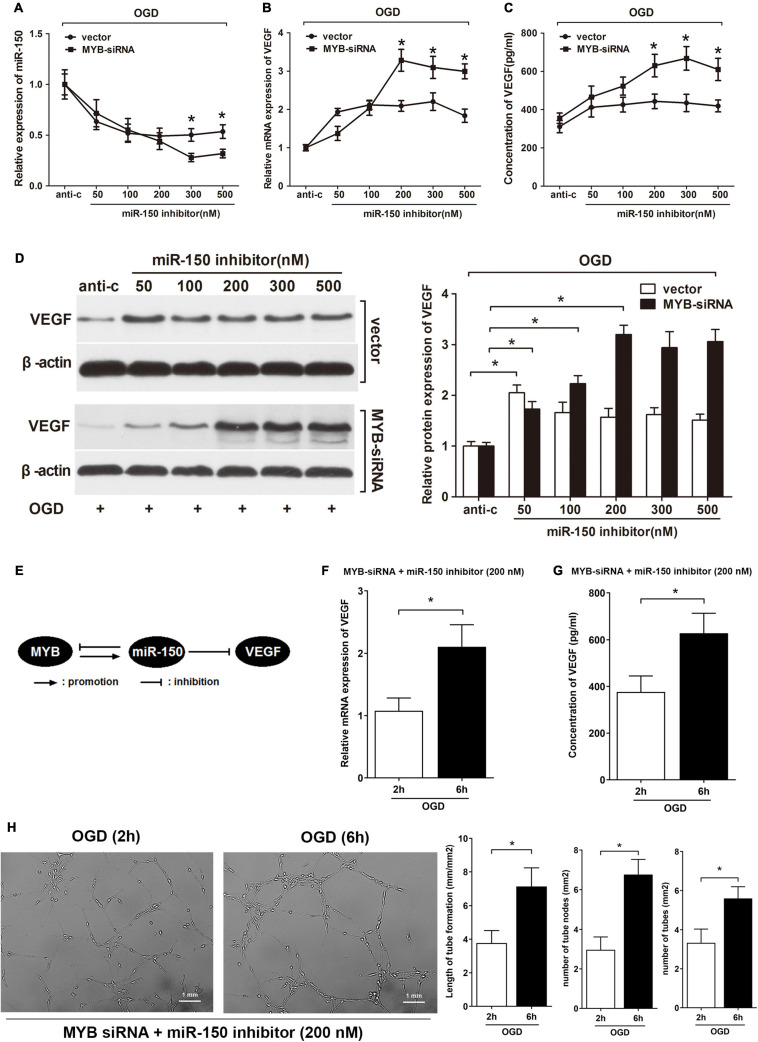
Repression of MYB is required for miR-150 inhibitor mediated further enhancement of VEGF in BMVECs during OGD. **(A)** BMVECs were co-transfected with different levels of miR-150 inhibitors and MYB siRNA or vector. Then BMVECs were exposed to OGD for 6 h and re-perfused for 24 h. qRT-PCR analysis of expression levels of **(A)** miR-150 and **(B)** VEGF mRNA was carried out in treated BMVECs. Data showed further repression of miR-150 whereas significant enhancement of VEGF mRNA with higher levels (>200 nM) of miR-150 inhibitors in MYB siRNA transfected group compared to vector group. **(C)** ELISA was performed to measure the concentration of VEGF. BMVECs co-transfected with MYB siRNA and higher levels (>200 nM) of miR-150 inhibitors produced more VEGF according to the results. **(D)** Western blotting was performed to detect protein expression of endogenous VEGF in BMVECs. A delayed higher plateau was observed in MYB deficiency group. The VEGF/β-actin ratio of miR-150 inhibitor control transfected group was set to 1.0, then values of other groups were calculated accordingly. **(E)** Schematic diagram showing that MYB and miR-150 form a negative feedback loop to regulate expression of VEGF in BMVECs treated with OGD. Then BMVECs treated with MYB-siRNA and miR-150 inhibitor (200 nM) were exposed to OGD for different time periods (2 and 6 h). Expression of VEGF was detected by **(F)** qRT-PCR and **(G)** ELISA. Matrigel assay was performed to calculate the tube length and number of BMVECs **(H)**. Data are presented as mean ± SD, *n* = 5 of three independent experiments. *p < 0.05 versus the control group.

Furthermore, we investigated the combined effect of miR-150 and MYB on VEGF expression in BMVECs under OGD. A rescue experiment was conducted by transfecting different levels of miR-150 inhibitors and MYB siRNA into BMVECs subjected to OGD for 6 h. In this case, knockdown of MYB probably further down-regulated miR-150 while enhanced VEGF expression. As expected, miR-150 expression, detected by qRT-PCR showed a continuous decrease until the level of miR-150 inhibitors was increased to 300 nM. Whereas, transfection with vector attenuated the reduction of miR-150, miR-150 expression did not further decrease when the level of miR-150 inhibitors was increased beyond 50 nM (Figure 4A). Since increased mRNA level of VEGF was related to inhibition of miR-150, repression of MYB also provided a more durable enhancement of VEGF mRNA until the level of miR-150 inhibitors was increased to 200 nM as compared to anti-miR control (anti-c) (Figure 4B). Moreover, ELISA showed that VEGF expression in BMVECs transfected with MYB siRNA continued to rise until the level of miR-150 inhibitors was increased to 300 nM whereas less VEGF expression was observed in BMVECs transfected with vector where VEGF did not increase further after the level of miR-150 inhibitors was increased beyond 50 nM (Figure 4C). Consistent with the elevation of VEGF mRNA, western blotting identified that suppression of protein expression in BMVECs by miR-150 was rescued by the addition of MYB siRNA (Figure 4D). Taken together, the addition of MYB siRNA along with miR-150 inhibitors led to further down-regulation of miR-150 and significant enhancement of VEGF in BMVECs under OGD, indicating that MYB and miR-150 form a negative feedback loop to regulate expression of VEGF in BMVECs treated with OGD (Figure 4E). Expression of VEGF reach the maximum when we break the feedback loop of miR-150 and MYB with MYB-siRNA and miR-150 inhibitor (200 nM) under OGD (6 h). Then we made a summary graph comparing the early (2 h) to late-response (6 h) in terms of RNA/protein levels (Figures 4F,G) of all these three actors (MYB, mIR150, and VEGF) and performed a Matrigel assay to show the effect of VEGF on the tube formation (Figure 4H).

## Discussion

Mounting number of experiments have been carried out regarding regulation of VEGF, the most important regulator in angiogenesis (Melincovici et al., 2018; Shim and Madsen, 2018; Apte et al., 2019), while the fact we should pay attention to is that, VEGF not only takes part in the restoration of blood supply after stoke, but also contributes to increased vascular permeability, leading to the destruction of BBB (Zhang et al., 2017; Geiseler and Morland, 2018). Therefore, appropriate amount of VEGF is essential for angiogenesis after ischemia. A variety of miRNAs could act as promoters or inhibitors of angiogenesis through targeting different factors including VEGF (Dang et al., 2013; Chen et al., 2019; Lu et al., 2020). However, very few studies have focused on the regulation of VEGF amount. We previously proved that miR-150 acts as an anti-angiogenic-miR by targeting the binding sites in 3′UTR of VEGF mRNA and extended the regulatory network of VEGF (He et al., 2016). Repression of miR-150 by miR-150 inhibitor was found to up-regulate the expression levels of VEGF in BMVECs. We next expected further enhancement of VEGF in the presence of higher levels of miR-150 inhibitors. Intriguingly, present results verified that BMVECs transfected with large doses of miR-150 inhibitors did not exhibit further enhancement of VEGF compared to the normal amount while expression of miR-150 also showed no more alteration. This finding convinced us that a novel factor existed that attenuates down-regulation of miR-150 by inhibitors, thus maintaining the amount of VEGF. Host organisms always hold various ways to maintain homeostasis of different factors. Many negative feedback loops occur in miRNA mediated gene regulation to maintain a balanced state (Masalha et al., 2018). For instance, miR-34a and p53 feedback promotes lung epithelial injury (Shetty et al., 2017), miR-301a/Cip2a feedback loop modulates cell proliferation in breast cancer (Yin et al., 2019). Since VEGF is also the direct target of miR-150, we consequently employed a test to determine whether VEGF was the very negative regulator to maintain a balance with miR-150. Present results excluded our hypothesis and the novel regulator was further investigated.

Our study has identified miR-150 as a potent regulator of cerebral angiogenesis that acts by modulating endothelial cells behaviors like migration, proliferation. in ischemia induced retinopathy model of mouse, miR-150 was reported to inhibit retinal angiogenesis as well (Shi et al., 2016). MiR-150 could also enhance the migration, tube formation, homing, thrombus recanalization and resolution ability of rat marrow derived endothelial progenitor cells (Wang et al., 2014). In addition, MYB was a canonical predicted target of miR-150, which impacted on proliferation of tumor cells in cooperation with miR-150 (Sun et al., 2019). Chen-Yu Zhang has showed that miR-150 from monocytic cell line THP-1 inhibited endothelial cells migration by targeting MYB, as a functional consequence, also modulated angiogenesis (Zhang et al., 2010). Moreover, MYB was demonstrated to promote VEGF expression via DNA-binding independent transcription (Lutwyche et al., 2006) as we have validated the promotion effect of MYB on VEGF in BMVECs under normal condition, indicating a probable direct pathway for MYB to regulate expression of VEGF, making a complicated crosstalk between each other. According to the reports above, miR-150 and MYB exhibited similar functions like cells migration, proliferation, angiogenesis along with VEGF production, presenting a possibility for interaction between these two regulators. We carried a series of tests to find that repression of MYB led to impairment of tube formation by brain BMVECs exposed to OGD, but in the meantime, growing expression of VEGF could be observed under the same condition. This was due to decreased expression of miR-150 caused by MYB reduction since level of VEGF was inversely related to miR-150 expression which we mentioned above. MYB could act as a regulator to control VEGF expression via miR-150. As expected, we found MYB as a promoter of miR-150 which also directly target MYB, forming a negative feedback loop with miR-150 to maintain a balance between each other, resulting in moderate alteration of VEGF expression. We also noticed that when BMVECs was cultured under normoxia condition (control), knockdown of MYB led to reduction of VEGF amount while miR-150 inhibitors rescued this condition, it was probably due to more effective induction of VEGF by suppressing miR-150 versus VEGF activation by MYB. Elevated quantity of VEGF generated by MYB inhibition mediated decrease of miR-150 exceeded reduced amount of VEGF by MYB repression. Direct relationship between MYB and VEGF need to be further explored.

Expression levels of VEGF were found to increase in BMVECs exposed to OGD. Consistent with the results from other kinds of cells under OGD condition, enhancement of VEGF may be partly due to simultaneous down-regulation of miR-150 and up-regulation of MYB both of which induce VEGF expression. Moreover, when we employed MYB siRNA transfection to decrease MYB expression under OGD, miR-150 expression was further down-regulated, whereas up-regulation of VEGF was observed again, suggesting that increased MYB served primary function as a positive regulator to slowdown the decline of miR-150 under OGD while its effect on VEGF induction did not play the main role. Further, BMVECs were co-transfected with different levels of miR-150 inhibitors and MYB siRNA in response to OGD. As a result of inhibition of MYB, the balanced state between miR-150 and MYB was broken, leading to further enhancement of VEGF after addition of higher level of miR-150. What also noticed us is the prediction of multiple downstream angiogenic factors targeted by miR-150, indicating that MYB may be not be the sole regulator conversely modulating miR-150. Other factors that form feedback loops with miR-150 for regulation of VEGF probably exist as well. There were several limitations within our study including that the study was conducted only *in vitro*. Practical *in vivo* experiments need to be carried out to explore the exact levels of VEGF expression controlled by this feedback loop. And the underlying mechanism of miR-150 promotion by MYB was not known, further work will be required in this aspect.

Taken together, we identified MYB as a potent regulator of VEGF expression via miR-150 in BMVECs of rats after OGD. During this process, miR-150 acted as a bridge between MYB and VEGF and was predicted to target MYB, while MYB exerted its primary function by increasing miR-150 along with an additive effect to maintain VEGF when BMVECs were exposed to OGD condition. Consequently, miR-150 and MYB formed a negative regulatory feedback loop to keep a balanced state, resulting in the homeostatic expression of VEGF. Furthermore, when MYB was inhibited to break this balance, exogenous miR-150 inhibitors further down-regulated miR-150 in BMVECs exposed to OGD, as a result, high levels of miR-150 inhibitors induced significantly elevated expression of VEGF in comparison with vector groups. Collectively, we showed that an endogenous negative feedback loop consisting of miR-150 and MYB regulates the expression level of VEGF.

## Data Availability Statement

The original contributions presented in the study are included in the article/supplementary material, further inquiries can be directed to the corresponding author/s.

## Author Contributions

SZ, AC, and XC performed the experiment, analyzed the data, and wrote the manuscript. SZ and AC performed the experiment, analyzed the data, and prepared the images. AC and XC designed and conducted the research, interpreted the data, and wrote the manuscript. All authors contributed to the article and approved the submitted version.

## Conflict of Interest

The authors declare that the research was conducted in the absence of any commercial or financial relationships that could be construed as a potential conflict of interest.
